# Automated antigen assays display a high heterogeneity for the detection of SARS-CoV-2 variants of concern, including several Omicron sublineages

**DOI:** 10.1007/s00430-023-00774-9

**Published:** 2023-08-10

**Authors:** Andreas Osterman, Franziska Krenn, Maximilian Iglhaut, Irina Badell, Andreas Lehner, Patricia M. Späth, Marcel Stern, Hanna Both, Sabine Bender, Maximilian Muenchhoff, Alexander Graf, Stefan Krebs, Helmut Blum, Timo Grimmer, Jürgen Durner, Ludwig Czibere, Christopher Dächert, Natascha Grzimek-Koschewa, Ulrike Protzer, Lars Kaderali, Hanna-Mari Baldauf, Oliver T. Keppler

**Affiliations:** 1 Max von Pettenkofer Institute and Gene Center, Virology, National Reference Center for Retroviruses, LMU München, Munich, Germany; 2 German Center for Infection Research (DZIF), Partner Site, Munich, Germany; 3 COVID‑19 Registry of the LMU Munich (CORKUM), University Hospital, LMU München, Munich, Germany; 4 Laboratory for Functional Genome Analysis, Gene Center, LMU München, Munich, Germany; 5 Department of Psychiatry and Psychotherapy, Klinikum Rechts der Isar, School of Medicine, Technical University of Munich, Munich, Germany; 6 Labor Becker MVZ GbR, Munich, Germany; 7 Department of Conservative Dentistry and Periodontology, University Hospital, LMU München, Munich, Germany; 8 Institute of Virology, Technical University of Munich/Helmholtz Zentrum München, Munich, Germany; 9 Institute of Bioinformatics, University Medicine Greifswald, Greifswald, Germany

**Keywords:** SARS-CoV-2, Variant of concern, Omicron, BA.1, BA.2, BA.5, BQ.1, XBB.1.5, Sublineage, Automated antigen tests, Nucleocapsid protein, Diagnostic test, Sensitivity, Specificity

## Abstract

Diagnostic tests for direct pathogen detection have been instrumental to contain the severe acute respiratory syndrome coronavirus type 2 (SARS-CoV-2) pandemic. Automated, quantitative, laboratory-based nucleocapsid antigen (Ag) tests for SARS-CoV-2 have been launched alongside nucleic acid-based test systems and point-of-care (POC) lateral-flow Ag tests. Here, we evaluated four commercial Ag tests on automated platforms for the detection of different sublineages of the SARS-CoV-2 Omicron variant of concern (VoC) (B.1.1.529) in comparison with “non-Omicron” VoCs. A total of 203 Omicron PCR-positive respiratory swabs (53 BA.1, 48 BA.2, 23 BQ.1, 39 XBB.1.5 and 40 other subvariants) from the period February to March 2022 and from March 2023 were examined. In addition, tissue culture-expanded clinical isolates of Delta (B.1.617.2), Omicron-BA.1, -BF.7, -BN.1 and -BQ.1 were studied. These results were compared to previously reported data from 107 clinical “non-Omicron” samples from the end of the second pandemic wave (February to March 2021) as well as cell culture-derived samples of wildtype (wt) EU-1 (B.1.177), Alpha VoC (B.1.1.7) and Beta VoC (B.1.351)). All four commercial Ag tests were able to detect at least 90.9% of Omicron-containing samples with high viral loads (Ct < 25). The rates of true-positive test results for BA.1/BA.2-positive samples with intermediate viral loads (Ct 25–30) ranged between 6.7% and 100.0%, while they dropped to 0 to 15.4% for samples with low Ct values (> 30). This heterogeneity was reflected also by the tests’ 50%-limit of detection (LoD50) values ranging from 44,444 to 1,866,900 Geq/ml. Respiratory samples containing Omicron-BQ.1/XBB.1.5 or other Omicron subvariants that emerged in 2023 were detected with enormous heterogeneity (0 to 100%) for the intermediate and low viral load ranges with LoD50 values between 23,019 and 1,152,048 Geq/ml. In contrast, detection of “non-Omicron” samples was more sensitive, scoring positive in 35 to 100% for the intermediate and 1.3 to 32.9% of cases for the low viral loads, respectively, corresponding to LoD50 values ranging from 6181 to 749,792 Geq/ml. All four assays detected cell culture-expanded VoCs Alpha, Beta, Delta and Omicron subvariants carrying up to six amino acid mutations in the nucleocapsid protein with sensitivities comparable to the non-VoC EU-1. Overall, automated quantitative SARS-CoV-2 Ag assays are not more sensitive than standard rapid antigen tests used in POC settings and show a high heterogeneity in performance for VoC recognition. The best of these automated Ag tests may have the potential to complement nucleic acid-based assays for SARS-CoV-2 diagnostics in settings not primarily focused on the protection of vulnerable groups. In light of the constant emergence of new Omicron subvariants and recombinants, most recently the XBB lineage, these tests’ performance must be regularly re-evaluated, especially when new VoCs carry mutations in the nucleocapsid protein or immunological and clinical parameters change.

## Introduction

The detection and quantification of SARS-CoV-2 RNA by nucleic acid–amplification testing (NAT, e.g., RT-PCR) represent the methodological gold standard in diagnostic laboratories. To add to the repertoire of quality-controlled, laboratory-based SARS-CoV-2 testing from respiratory material, automated, quantitative SARS-CoV-2 antigen (Ag) assays were introduced in late 2020. While a considerably lower sensitivity and variable specificity of nucleocapsid Ag-based detection systems has been documented [1–5], both lateral flow RATs, primarily used in a POC setting, and automated Ag assays may contribute to a clinical and outbreak management, especially during periods of high incidence [6–10].

Evidence is accumulating that SARS-CoV-2 variants of concern (VoCs), which have been constantly evolving during the COVID-19 pandemic, underlie an increasing variability in the recognition by nucleocapsid-based detection systems [11–17]. Although several studies have investigated the sensitivity of automated Ag tests as a function of Ct values, there is little data on the influence of variants or especially Omicron-VoC subvariants [5, 18–23]. Thus, previous validations may not be predictive for the performance of RATs or automated Ag assays for detecting individuals infected with emerging VoCs [11, 12, 21, 24]. Conceivably, for hospitals, elderly homes or schools, automated quantitative SARS-CoV-2 Ag assays may provide a useful alternative to PCR testing, facilitating a medium-throughput screening at lower costs of larger cohorts of patients, employees or students in a relatively short period of time. Especially for the setting of hospitals and elderly homes it is critical though to use assays validated to perform at high sensitivity and ideally also high specificity to protect vulnerable individuals. We and others have recently evaluated automated SARS-CoV-2 Ag tests for the detection of SARS-CoV-2, including different VoCs [1, 6–10, 17, 21, 25–37]. Their specificities ranged between 92.6 and 100% [1, 6–10, 17, 21, 25–37] with those performing at levels > 97% fulfilling the WHO ‘s specificity criterion for these types of diagnostic assays [38]. Sensitivities of automated SARS-CoV-2 Ag assays were reported to vary drastically between test systems, ranging between 17.8% and 98.8% [1, 6–10, 17, 21, 25–37].

Ever since its appearance in November 2021 [39] the Omicron (B.1.1.529) VoC and its subvariants have dominated the COVID-19 pandemic. Recently, however, a number of additional Omicron-BA.2 subvariants (BN.1, BJ.1, BA.2.75), BA.5 subvariants (BF.7, BQ.1.1) and XBB recombinants have been emerging [40]. The Omicron subvariants BA.1, BA.2, BA.4 and BA.5 are characterized by many more mutations in the spike protein compared to previous VoCs, but also unique mutations in the nucleocapsid protein [41, 42] (Table 1). These mutations have in part been associated with enhanced viral infectivity and more pronounced humoral escape [43–45]. In light of novel nucleocapsid mutations that may affect the binding of diagnostic test antibodies, or recently proposed changes in anti-SARS-CoV-2 immunity in the general population, or potentially altered ratios of nucleocapsid protein to viral RNA on the respiratory mucosa [24, 46], it is pertinent to regularly re-evaluate Ag-based tests for performance characteristics, in particular their sensitivity to detect emerging VoCs.

**Table 1:** Characteristics of the three groups of specimen (historical “Non-Omicron” [32], Omicron 2022 (BA.1/BA.2) and Omicron 2023 (BA.2/BA.5)), respiratory swab sampling and virus expansion

	Non-Omicron (historical data*)	Omicron 2022 (BA.1 and BA.2)	Omicron 2023 (BA.2 and BA.5, including BQ.1, XBB.1.5 and other subtypes)
Collecting sites	LMU Klinikum, three teaching hospitals of the LMU Munich	Regional hospitals, COVID-19 testing centers, nursing homes and primary care physicians’ offices	Regional hospitals, COVID-19 testing centers, nursing homes and primary care physicians’ offices
Sampling period	February 1^st^ to March 1^st^, 2021	February 22^nd^ to March 3^rd^, 2022	March 1st to March 15th
Number of swabs	SARS-CoV-2-PCR-positive: 107 SARS-CoV-2-PCR-negative: 303	SARS-CoV-2-PCR-positive: 101 (Omicron-BA.1: 53, -BA.2: 48)	SARS-CoV-2-PCR-positive: 102 (Omicron BQ.1: 23; XBB1.5: 39, other subtypes: 40
Type of swabs	IMPROVIRAL™ with 3 ml Viral Preservative Medium (VPM) (Improve Medical, Guangzhou, Republic of China) CITOSWAB® with 3 ml Viral Transport Medium (VTM) (Citotest Scientific Co.,Ltd, Jiangsu, Republic of China)	Sigma-Transwab® with 1 or 2 ml Amies Transport Medium (Medical Wire & Equipment Co Ltd; Corsham, UK)	Sigma-Transwab® with 1 or 2 ml Amies Transport Medium (Medical Wire & Equipment Co Ltd; Corsham, UK)
Storage	2–8 °C for up to 24 h (ECLIA: up to 1 week after inactivation at -20 °C)	2–8 °C for up to 24 h (CLEIA: up to 1 week at -20 °C)	2–8 °C for up to 24 h (CLEIA: up to 30 days at -20 °C)
SARS-CoV-2 RNA extraction/detection/quantification	Maxwell RSC Viral Total Nucleic Acid Purification Kit Center for Disease Control (CDC) protocol (N1 reaction) Seegene Allplex 2019-nCoV Assay Roche Cobas SARS-CoV-2 Test Xpert Xpress SARS-CoV-2/Flu/RSV	Screening: „Munich Extraction Protocol “ [55] RIDA®GENE SARS-CoV-2 RUO assay (R-Biopharm) Quantification: Roche Cobas SARS-CoV-2 Test	Screening: „Munich Extraction Protocol “ [55] RIDA®GENE SARS-CoV-2 RUO assay (R-Biopharm) Quantification: Roche Cobas SARS-CoV-2 Test
Genotyping	Full genome next generation sequencing (ARTIC network nCoV-2019 sequencing protocol v2)	COVID-19 direct RT-PCR kit (FRIZ Biochem GmbH)	COVID-19 direct RT-PCR kit (FRIZ Biochem GmbH) followed by full genome next generation sequencing (ARTIC network nCoV-2019 sequencing protocol v4.1)
Cell culture isolate	wt EU-1 (B.1.177); Alpha VoC (B.1.1.7); Beta VoC (B.1.351)	Omicron (B.1.529; Omicron-BA.1); Delta (B.1.617.2)	Omicron (BQ.1.1; BF.7; BN.1)
Cell culture expansion	Challenge: Caco-2 cells Propagation: Caco-2 or Vero-E6 cells	Challenge and propagation: Vero-E6 cells	Challenge and propagation: Vero-E6 cells

^*^[32]

Here, we re-evaluated four commercial automated Ag tests, i.e., CLEIA from Fujirebio Inc., CLIA from DiaSorin S.p.A., ELISA from Euroimmun Medizinische Labordiagnostika AG and the ECLIA assay from Roche Diagnostics GmbH, for their performance in samples containing various Omicron subvariants. Recently, we reported a variable detection of Omicron-BA.1 and -BA.2 by five different SARS-CoV-2 RATs [47], indicating that the identification of individuals infected with these subvariants based on nucleocapsid detection may pose a particular challenge. In the current report, results from four automated Ag tests were compared to these assays’ performance for the detection of “non-Omicron” SARS-CoV-2-containing samples from our previous study [32]. Besides respiratory samples, we also evaluated cell culture-expanded clinical isolates of VoCs Delta and several Omicron subvariants to directly address potential performance issues based on antibody binding to nucleocapsid proteins carrying several mutations.

## Materials and methods

### Respiratory swabs

In the periods February 22nd to March 3rd 2022 and March 1st to March 15th 2023, swabs from the upper respiratory tract were collected by healthcare professionals and sent to Labor Becker MVZ GbR in Munich, Germany, a diagnostic laboratory that receives samples from regional hospitals, COVID-19 testing centers, nursing homes, and family practices. SARS-CoV-2 RNA was detected by real-time RT-qPCR and identified as Omicron-BA.1, -BA.2 or -BA.5 by typing PCR and included in the study’s Omicron cohort. The differentiation of the subgroups in the second study phase in 2023 was subsequently carried out via whole-genome sequencing. All samples with a measurable crossing point/cycle threshold (Cp/Ct) value quantified by quantitative RT-PCR under accredited conditions less than 24 h after specimen collection were considered “SARS-CoV-2-positive”. All samples from the first study period were randomly included in the study, depending on the availability of a sufficient sample volume. During the second testing period, approximately the same number of samples with comparable virus loads were selected to facilitate comparability of results. No information about vaccination status of individuals, previous infections, presenting symptoms or clinical course was available. For this study, flocked swabs Sigma-Transwab® with 1 or 2 ml Amies Transport Medium were collected. Patient specimens in liquid transport medium with the potential for denaturation were excluded from the study. Original respiratory swabs and transport media were stored at 2–8 °C for up to 24 h, until samples were inactivated and SARS-CoV-2 Ag testing was performed. A total of 203 PCR-positive (BA.1: 53, BA.2: 48; BQ.1: 23; XBB.1.5: 39, other Omicron subtypes: 40) respiratory samples were analyzed within the Omicron cohort. The "non-Omicron" cohort served as an informative reference consisting of historical data from 107 SARS-CoV-2-PCR-positive and 303 PCR-negative respiratory samples collected by the same protocol during the period February 1st to March 1st, 2021 at the LMU Klinikum and three teaching hospitals of LMU Munich and analyzed at the Max von Pettenkofer Institute [32] (Table 1).

### Automated SARS-CoV-2 antigen tests

All tests were performed according to the manufacturer's instructions. The automated Ag tests used in the current study are identical to those from our previous evaluation [32]. Despite the prospective study design, not all manufacturer's recommendations regarding swab kit/transport media and sample storage conditions could be followed. The Lumipulse G SARS-CoV-2 Ag ("CLEIA") from Fujirebio Inc (Tokyo, Japan) is a chemiluminescence enzyme immunoassay for the quantitative measurement of SARS-CoV-2 Ag in samples based on CLEIA technology [48], a specific two-step immunoassay on the LUMIPULSE G system [49]. As recommended by the manufacturer, inactivated samples were centrifuged at 2,000 × g for 5 min prior to testing in this study. In our previous characterization study, this assay showed the lowest limit of detection and a wide dynamic range [32]. In this study, in contrast to our previous study, the swab samples were stored for up to 30 days at -20 °C prior to inactivation, which is in line with recommendations by the manufacturer. The LIAISON® SARS-CoV-2 Ag ("CLIA") assay from DiaSorin S.p.A. (Saluggia, Italy) utilizes a direct, two-step sandwich chemiluminescence immunoassay (CLIA) for the quantitative determination of SARS-CoV-2 in nasal swabs or nasopharyngeal swabs [50]. The manufacturer announced at the beginning of the Omicron wave that the LIAISON® SARS-CoV-2-Ag assay uses polyclonal antibodies and is, therefore, less prone to performance loss due to mutations in the nucleocapsid protein. In the package leaflet, the manufacturer states that in-house testing with recombinant Omicron nucleocapsid Ag and ten nasopharyngeal swabs proves that Omicron is recognized with unchanged sensitivity [50]. The manufacturer specifies in the package insert that nasopharyngeal swabs in VTM/UTM should only be stored for 12 h at 2–8 °C prior to transferring the sample into the inactivation buffer, which could not be fulfilled in this study (see above) [50]. The Elecsys SARS-CoV-2 Antigen ("ECLIA") assay from Roche Diagnostics GmbH (Mannheim, Germany) uses an antibody sandwich principle (anti-SARS-CoV-2 monoclonal antibodies (mouse and rabbit)) in an electrochemiluminescence immunoassay (ECLIA) to detect SARS-CoV-2 nucleocapsid protein in nasopharyngeal and oropharyngeal swab specimens [51]. The manufacturer states on its homepage that this assay reliably detects all currently circulating variants including Omicron [52]. The SARS-CoV-2 Ag ELISA ("ELISA") from Euroimmun Medizinische Labordiagnostika AG (Lübeck, Germany) is a semi-quantitative enzyme-linked immunosorbent assay (ELISA) for the in vitro detection of SARS-CoV-2 nucleocapsid from nasopharyngeal swabs [53]. The manufacturer states, that based on initial analyses of the available sequences of Omicron, the detection of nucleocapsid is not affected [54]. Shortly after completion of the experimental phase of our study, this test was withdrawn from the market by the manufacturer.

### Quantitative viral load determination

Prior to testing the Omicron swab specimens for the presence of nucleocapsid protein, the amount of SARS-CoV-2 RNA present was determined in the accredited routine diagnostic laboratories of the Max von Pettenkofer Institute using the Roche Cobas SARS-CoV-2 E-Gen reaction on a Cobas 6800 system. Details on the reference "Non-Omicron" cohort and the evaluation of SARS-CoV-2 PCR-negative samples in these automated assays have been reported [32] and are summarized in Table 1. Viral load conversion was performed using an in-house standard curve as previously described [24]. This quantification was performed not later than 24 h after collection of the swab. In general, the calculations for quantification do not consider variability between separate quantitative RT-PCR runs. However, since this variability applies to all study groups, they do not affect the interpretation of the results in this study. The initial PCR screening of the Omicron sample cohort was performed by the Labor Becker MVZ GbR using the „Munich Extraction Protocol “ [55].

### SARS‑CoV‑2 genotyping

For the Omicron cohort, PCR screening was performed using the “Munich Extraction Protocol” [55], followed by variant-specific PCR analyses (modified version of the COVID-19 direct RT-PCR kit (FRIZ Biochem GmbH, Neuried, Germany) to identify samples with BA.1, BA.2, or BA.5. These investigations were performed under routine diagnostic laboratory conditions at Labor Becker MVZ GbR on freshly collected samples. Typing and characterization of the “non-Omicron” sample cohort was performed using next-generation sequencing as reported [32].

### Analysis of SARS-CoV-2 BA.2 and BA.5 sublineages by whole-genome sequencing

During the second study phase in March 2023, due to the large number of variants that have emerged in the meantime, the variants initially assigned as BA.2 or BA.5 by means of variant-specific PCR were further analyzed using whole-genome sequencing and afterwards subdivided into subgroups as described above. According to the sequencing protocol v4.1 of the ARTIC network nCoV-2019, amplicon pools covering the SARS-CoV-2 genome were generated. These were analyzed using the Artic bioinformatics protocol, the principle of which has already been described in previous publications [56, 57]. The consensus sequences and associated sample metadata have been uploaded to the GISAID repository.

### Propagation of SARS‑CoV‑2 from primary patient material

Clinical isolates of Delta, Omicron-BA.1, -BF.7, -BN.1 and -BQ.1 from respiratory material were propagated on Vero-E6 cells (Table 1) and characterized as reported [24]. Expanded stocks of wt EU-1, Alpha, Beta, Delta and Omicron subvariants were analyzed by whole genome sequencing (B.1.177: GISAID EPI ISL: 466,888; B.1.1.7; GISAID EPI ISL: 2,094,739; B.1.351; GISAID EPI ISL: 1,752,394; B.1.617.2: GISAID 3233464; B.1.1.529: GISAID 7808190; BQ.1: GISAID EPI ISL 15812431; BF.7: GISAID EPI ISL 15825638; BN.1: GISAID EPI ISL 16909949) and RNA copies per mL were determined as the mean from technical triplicates using an in-house PCR calibrated with reference material with a defined copy number distributed by INSTAND e.V.

### Statistical analyses

Statistical analysis was performed in R version 4.1.2, using the pROC package to perform receiver operator characteristic curve (ROC curve) analysis [58]. Binomial confidence intervals for sensitivities and specificities were computed using the Wilson score interval. To further analyze analytical sensitivities, we used logistic regression, with viral loads as independent and test outcomes as the dependent variable, yielding detection probabilities for each viral load level.

## Results

**Fig. 1:**
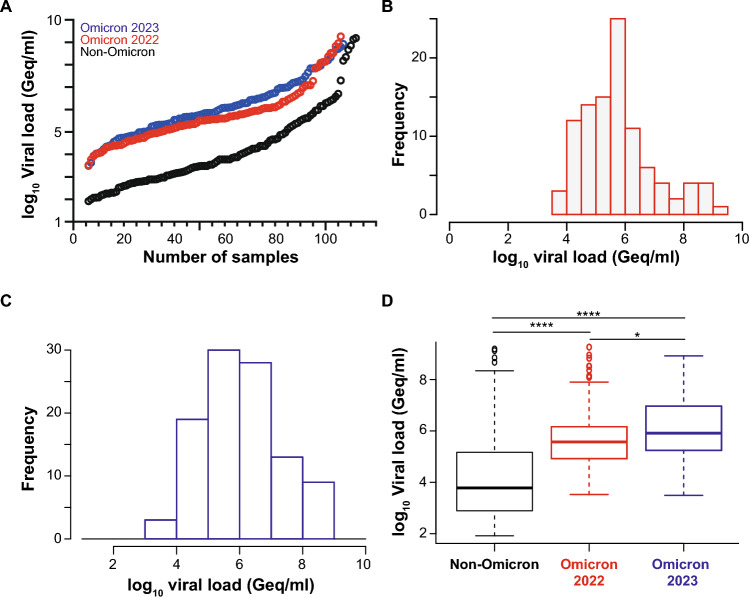
SARS-CoV-2 viral load distribution of respiratory samples included in the study. **A** Shown is the log10 viral load (Geq/ml) of 203 SARS-CoV-2-positive (101 Omicron 2022; red and 102 Omicron 2023; blue) versus 107 SARS-CoV-2-positive (“non-Omicron”; black [32]) patient samples, sorted by ascending magnitude from left to right. Each dot indicates one patient and the sample’s ID is indicated. **B** and **C** Depicted is the histogram of the viral load distribution by categorization of samples into defined log10 viral load value ranges. Each bar indicates the number of samples in the respective viral load range**; B** for Omicron 2022 samples (red), **C** for Omicron 2023 samples (blue)**. D** The horizontal line in the box plots shows the median of the samples shown in A, bound between upper and lower quartiles, and whiskers between minimum and maximum are indicated. *p < 0.05, ****p < 0.000005 by Wilcoxon rank sum test with continuity correction and by two-sample Kolmogorov–Smirnov test

We previously reported an acceptable specificity of the four automated Ag tests for SARS-CoV-2 evaluated in the current study, ranging between 97.0 and 99.7% [32]. Here, we tested the analytical sensitivity of these automated Ag test systems to detect different dominant Omicron subvariants, specifically BA.1, BA.2, BQ.1 and XBB.1.5 as well as other minor subvariants. We included 203 SARS-CoV-2 PCR-positive nasopharyngeal swabs, of which 53 were classified as BA.1, 48 as BA.2, 23 as BQ.1, 39 as XBB.1.5 and 40 as other minor subvariants, respectively, and compared those to the 107 “non-Omicron” SARS-CoV-2 swabs, results for which were reported recently [32]. In contrast to the initial “non-Omicron” data set with a viral load range of 83 to 1,548,572,803 Geq/ml (median: 6,045 Geq/ml), the swabs taken from patients with these Omicron subvariants showed considerably higher viral loads ranging from 3,361 to 1,824,858,018 Geq/ml for the Omicron subvariants from 2022 (median: 373,560 Geq/ml) and 3,129 to 832,225,970 Geq/ml for the Omicron subvariants from 2023 (median: 762,693 Geq/ml) (Fig. 1).

### Diagnostic sensitivity of automated antigen tests for Omicron subvariants

**Fig. 2:**
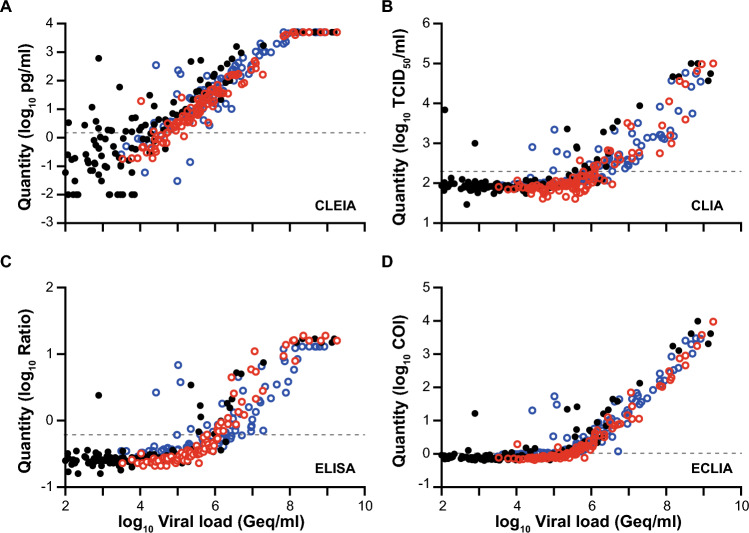
Analytical sensitivity of PCR-positive SARS-CoV-2 patient samples for quantitative SARS-CoV-2 Ag tests. **A** CLEIA from Fujirebio, **B** CLIA from Diasorin, **C** ELISA from Euroimmun and **D** ECLIA from Roche Diagnostics. Omicron 2022 samples are shown in red, Omicron 2023 samples in blue and data for “non-Omicron” samples retrieved from our previous study [32] are shown in black. The log10 of quantified samples were plotted against the log10 of the calculated viral loads. The grey dashed line indicates the cutoffs recommended by the manufacturers

We then compared the diagnostic sensitivity (Table 2) using the Ct/Cp value ranges of respiratory swabs for stratification, and analytical performance (Fig. 2) of the four automated SARS-CoV-2 Ag tests. To determine the analytical performance, we used the cutoffs for “positive” and “negative” scoring according to the manufacturers’ recommendations (CLEIA ≥ 1.34; CLIA ≥ 200; ELISA ≥ 0.60; ECLIA ≥ 1.00; grey dashed lines).

**Table 2:** Comparative evaluation of the diagnostic sensitivity of four automated SARS-CoV-2 Ag tests stratified for Ct/Cp value ranges for “non-Omicron”* and Omicron-containing respiratory samples

Assay align better below	Ct < 25 (%)	Ct 25–30 (%)	Ct > 30 (%)
*CLEIA*			
Non-Omicron*	100.0 (*n* = 11)	100.0 (*n* = 20)	32.9 (*n* = 76)
Omicron 2022	100.0 (*n* = 24)	96.6 (*n* = 58)	10.5 (*n* = 19)
* BA.1*	*100.0 (n* = *12)*	*100.0 (n* = *28)*	*15.4 (n* = *13)*
* BA.2*	*100.0 (n* = *12)*	*93.3 (n* = *30)*	*0.0 (n* = *6)*
Omicron 2023	*100.0 (n* = *38)*	*100.0 (n* = *52)*	*100.0 (n* = *12)*
* BQ.1*	*100.0 (n* = *11)*	*100.0 (n* = *11)*	*100.0 (n* = *1)*
* XBB.1.5*	*100.0 (n* = *13)*	*100.0 (n* = *20)*	*100.0 (n* = *6)*
* Other*	*100.0 (n* = *14)*	*100.0 (n* = *21)*	*100.0 (n* = *5)*
*CLIA*			
Non-Omicron*	100.0 (*n* = 11)	45.0 (*n* = 20)	1.3 (*n* = 76)
Omicron 2022	91.7 (*n* = 24)	6.9 (*n* = 58)	0.0 (*n* = 19)
* BA.1*	*91.7 (n* = *12)*	*7.1 (n* = *28)*	*0.0 (n* = *13)*
* BA.2*	*91.7 (n* = *12)*	*6.7 (n* = *30)*	*0.0 (n* = *6)*
Omicron 2023	*97.4 (n* = *38)*	*21.2 (n* = *52)*	*8.3 (n* = *12)*
* BQ.1*	*100.0 (n* = *11)*	*9.1 (n* = *11)*	*0.0 (n* = *1)*
* XBB.1.5*	*92.3 (n* = *13)*	*25.0 (n* = *20)*	*0.0 (n* = *6)*
* Other*	*100.0 (n* = *14)*	*23.8 (n* = *21)*	*20.0 (n* = *5)*
*ELISA*			
Non-Omicron*	100.0 (*n* = 11)	35.0 (*n* = 20)	1.3 (*n* = 76)
Omicron 2022	100.0 (*n* = 24)	12.1 (*n* = 58)	0.0 (*n* = 19)
* BA.1*	*100.0 (n* = *12)*	*14.3 (n* = *28)*	*0.0 (n* = *13)*
* BA.2*	*100.0 (n* = *12)*	*10.0 (n* = *30)*	*0.0 (n* = *6)*
Omicron 2023	*94.7 (n* = *38)*	*23.1 (n* = *52)*	*8.3 (n* = *12)*
* BQ.1*	*90.9 (n* = *11)*	*0.0 (n* = *11)*	*0.0 (n* = *1)*
* XBB.1.5*	*92.3 (n* = *13)*	*35.0 (n* = *20)*	*0.0 (n* = *6)*
* Other*	*100.0 (n* = *14)*	*23.8 (n* = *21)*	*20.0 (n* = *5)*
*ECLIA*			
Non-Omicron*	100.0 (*n* = 11)	35.0 (*n* = 19)	10.7 (*n* = 75)
Omicron 2022	100.0 (*n* = 24)	50.9 (*n* = 57)	5.3 (*n* = 19)
* BA.1*	*100.0 (n* = *12)*	*57.1 (n* = *28)*	*7.7 (n* = *13)*
* BA.2*	*100.0 (n* = *12)*	*44.8 (n* = *29)*	*0.0 (n* = *6)*
Omicron 2023	*100.0 (n* = *38)*	*92.2 (n* = *52)*	*41.7 (n* = *12)*
* BQ.1*	*100.0 (n* = *11)*	*90.9 (n* = *11)*	*0.0 (n* = *1)*
* XBB.1.5*	*100.0 (n* = *13)*	*89.5 (n* = *20)*	*66.7 (n* = *6)*
Other	*100.0 (n* = *14)*	*95.2 (n* = *21)*	*20.0 (n* = *5)*

The Omicron-containing samples are further subdivided into the 2022 (BA.1 and BA.2) and 2023 (XBB.1.5, BQ.1, other) study cohorts

^*^[32]

When stratifying the binary (“positive”, “negative”) test results according to the samples’ Ct/Cp value ranges, all four automated SARS-CoV-2 Ag tests were able to score true-positive results for at least 91.7% of Omicron-containing samples in 2022 with high viral loads (Ct < 25) (Table 2). The rates of true-positive results with intermediate viral loads (Ct 25–30) varied between 6.7% and 100.0%, while they decreased to 0 to 15.4% for samples with low Ct values (Ct > 30). For the Omicron subvariants in 2023, at least 90.9% of the samples were scored positive for high viral loads (Ct < 25), while those values varied markedly between 0 and 100% for both intermediate and low viral loads. Here, detection of BQ.1 by ELISA was worst among these categories. In contrast, the diagnostic detection of the “non-Omicron” samples was more sensitive for CLEIA, CLIA and ELISA, scoring positive in 35 to 100% (Ct 25–30) and 1.3 to 32.9% (Ct > 30) of cases, respectively (Table 2). Interestingly, Omicron-positive samples collected in 2023 for CLEIA and ECLIA were detected more readily compared to the Omicron samples collected in 2022 as well as the anecdotal cohort. It is of note that diagnostic sensitivities for BA.1, BA.2 and BQ.1 detection were comparable, although a trend towards a reduction in performance for the latter VoC was noted, while samples containing XBB.1.5 were superior in this respect. For the subsequent experiments and analyses, data for the Omicron subvariants from specimen sampled in 2022 and 2023 were combined, respectively, and compared to the “non-Omicron” samples. Most notably though, the diagnostic sensitivities of the four automated Ag test systems differed substantially among each other, in agreement with our previous report [32].

### Receiver operator characteristic analysis and evaluation of different cutoff values for Omicron sublineages

**Fig. 3:**
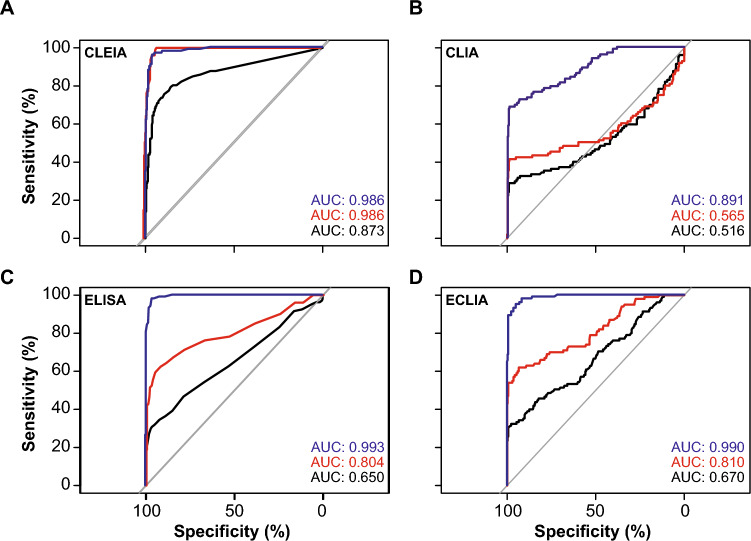
ROC analyses for quantitative SARS-CoV-2 Ag tests with **A** CLEIA, **B** CLIA, **C** ELISA and **D** ECLIA. The respective AUCs are depicted. Omicron 2022 samples are shown in red, Omicron 2023 samples in blue and data for “non-Omicron” samples retrieved from our previous study [32] are shown in black

We next evaluated receiver operator characteristic (ROC) curves of the four automated Ag tests, comparing their performance between Omicron 2022/2023- and “non-Omicron”-containing respiratory samples. CLEIA was best for the “non-Omicron” specimens with a calculated area under the curve (AUC) of 0.873 [32]. Using the Omicron 2022 (red) and 2023 (blue) samples for ROC evaluation, the AUC for CLEIA were even better, i.e., 0.986 (Fig. 3A). In comparison, the AUC for CLIA remained rather low with 0.565 for the 2022 samples while improving to 0.891 for the 2023 samples; for comparison, the “non-Omicron” AUC was 0.516 [32] (Fig. 3B). Interestingly, ELISA and ECLIA also showed high AUCs with 0.804 and 0.810 for the 2022 samples and 0.993 and 0.990 for the 2023 samples, respectively (“non-Omicron” ELISA: 0.650, “non-Omicron” ECLIA: 0.670 [32]) (Fig. 3C, D).

Based on the ROC analyses, we re-evaluated the cutoff values with our Omicron data set. We calculated the specificities and sensitivities, setting the WHO’s minimal criteria of > 80% sensitivity and > 97% specificity, respectively (Tables 3, 4). To reach a specificity of 97%, the sensitivity of CLEIA had to be lowered to 82.2% for the 2022 samples, while it was 91.0% for the 2023 samples (Table 3). Thus, CLEIA was always able to fulfill the WHO ‘s minimal criterion for sensitivity > 80% under conditions of acceptable specificity (Table 4). For the Omicron samples in 2022, CLIA’s sensitivity would stay rather unaffected compared to the optimal cutoff setting within the non-Omicron cohort upon adjustment of the specificity to 97% (41.6%, Table 3). On the contrary, specificity would be only 10.9% when increasing sensitivity to 80% (Table 4). For the Omicron samples in 2023, sensitivity improved to 68.6% for a specificity of 97%. To reach a sensitivity of 80%, the specificity for the Omicron samples in 2023 had to be markedly lowered to 71.3%. For ELISA, an increase in specificity to 97% with the Omicron samples in 2022 would thus not have a major impact on the sensitivity compared to the optimal non-Omicron-cutoff setting (52.6%, Table 3). Aiming for a sensitivity of 80% would, however, lower the specificity to 49.4% (Table 4). The Omicron samples from 2023 were superior in that respect: aiming for a specificity of 97%, the sensitivity was 94.3% and for a sensitivity of 80%, the specificity was 99.7%, respectively, fulfilling thereby the WHO’s minimal criteria. Compared to the optimal non-Omicron-cutoff setting, the sensitivity of ECLIA for the Omicron samples in 2022 would only be marginally reduced to 55.0% if a specificity of 97% should be achieved (Table 3). Aiming for a sensitivity of 80% would decrease the specificity to an unacceptable level of 49.5% (Table 4). Here, again, the Omicron samples in 2023 fulfilled the WHO’s minimal criteria: To reach a specificity of 97%, the optimal sensitivity was 91.1%, while the optimal specificity was 99.3% when setting the sensitivity to 80%.

**Table 3:** Cut-off-dependent calculation of extrapolated sensitivities using the WHO minimal criteria with specificity fixed at 97.0%

		Non-Omicron	Omicron 2022	Omicron 2023
Assay	Specificity (%)	Sensitivity (%)	Sensitivity (%)	Sensitivity (%)
CLEIA	97.0	52.3	82.2	91.0
CLIA	97.0	29.0	41.6	68.6
ELISA	97.0	29.9	52.6	94.3
ECLIA	97.0	32.4	55.0	91.1

**Table 4:** Cut-off-dependent calculation of extrapolated specificities using the WHO minimal criteria with sensitivity fixed at 80.0%

		Non-Omicron	Omicron 2022	Omicron 2023
Assay	Sensitivity (%)	Specificity (%)	Specificity (%)	Specificity (%)
CLEIA	80.0	85.0	97.4	98.3
CLIA	80.0	11.6	10.9	71.3
ELISA	80.0	28.8	49.4	99.7
ECLIA	80.0	30.0	49.5	99.3

### Analytical sensitivity of automated antigen tests for Omicron sublineages

**Fig. 4:**
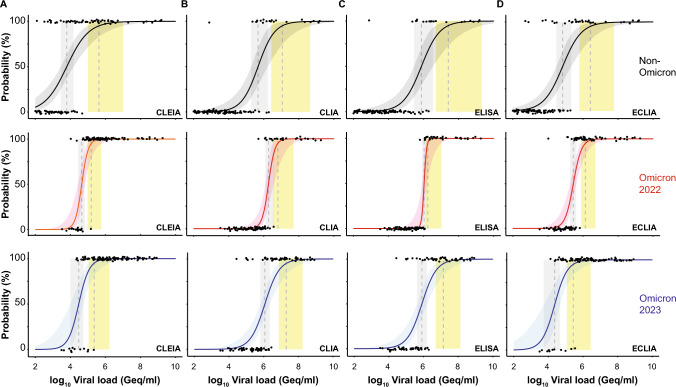
Limit of detection analyses of PCR-positive SARS-CoV-2 patient samples for quantitative SARS-CoV-2 Ag tests: **A** CLEIA,** B** CLIA,** C** ELISA, and **D** ECLIA. Omicron 2022 data set is shown in red, Omicron 2023 in blue and data for “non-Omicron” samples retrieved from [32] are shown in black. The log10 viral load of quantified samples on the x-axis was plotted against a positive (+ 1) or negative (0) test outcome on the y-axis. For readability of the figure, slight normal jitter was added to the y-values. Red/grey/blue curves show logistic regressions of the viral load on the test outcome; vertical dashed lines indicate log viral loads at which 50% (LoD50) and 95% (LoD95), respectively, of the samples are expected positive based on the regression results

Next, we calculated the 50% and 95% limits of detection (LoDs) based on a logistic regression model as reported recently [59]. The virus concentrations at which 50% (LoD50) and 95% (LoD95) detection rates were obtained for CLEIA with the Omicron samples in 2022 corresponded to 44,444 Geq/ml and 154,621 Geq/ml, while the ones for the Omicron samples in 2023 were 29,951 and 227,484 Geq/ml, respectively (“non-Omicron”: LoD50—6,181 Geq/ml, LoD95—422,689 Geq/ml [32]; Fig. 4A). The LoD50 and LoD95 of CLIA were 42-fold higher compared to CLEIA, respectively, and equaled 1,866,900 and 6,433,679 Geq/ml for the Omicron samples in 2022 (“non-Omicron”: LoD50—473,279 Geq/ml, LoD95—11,452,782 Geq/ml [32]; Fig. 4B). For the Omicron samples in 2023, the differences for LoD50 and LoD95 were comparable with 38-fold and 87-fold, respectively, which corresponded to 1,152,048 and 19,824,213 Geq/ml. ELISA yielded LoD50 and LoD95 with 1,069,098 and 1,742,957 Geq/ml for the Omicron samples in 2022, which were 24-fold and 11-fold higher compared to CLEIA, respectively (“non-Omicron”: LoD50—749,792 Geq/ml, LoD95—25,711,669 Geq/ml [32]; Fig. 4C). Those for the Omicron samples in 2023 were in a similar range with 27-fold and 61-fold higher values compared to CLEIA, respectively, corresponding to 794,733 and 13,895,613 Geq/ml. ECLIA was only sixfold and ninefold inferior to CLEIA for the Omicron samples in 2022 and resulted in 281,582 and 1,380,738 Geq/ml for LoD50 and LoD95, respectively (“non-Omicron”: LoD50—69,002 Geq/ml, LoD95—2,654,696 Geq/ml [32]; Fig. 4D). For the Omicron samples in 2023, LoD50 and LoD95 were comparable to CLEIA with 23,019 (LoD50) and 276,728 (LoD95) Geq/ml. It is of note that no statistically significant difference between the three cohorts, “non-Omicron”, Omicron-2022 as well as Omicron 2023, could be detected for all four automated Ag tests.

**Fig. 5:**
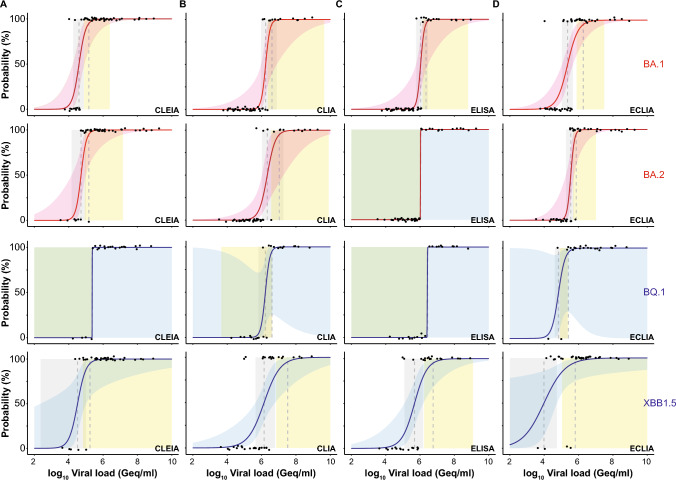
Limit of detection analyses of PCR-positive SARS-CoV-2 patient samples for quantitative SARS-CoV-2 Ag tests: **A** CLEIA,** B** CLIA,** C** ELISA, and **D** ECLIA. Omicron 2022 data set (subdivided in BA.1 and BA.2) is shown in red and Omicron 2023 (subtypes BQ.1 and XBB.1.5) samples are shown in blue. The log10 viral load of quantified samples on the x-axis was plotted against a positive (+ 1) or negative (0) test outcome on the y-axis. For readability of the figure, slight normal jitter was added to the y-values. Red/blue curves show logistic regressions of the viral load on the test outcome; vertical dashed lines indicate log viral loads at which 50% (LoD50) and 95% (LoD95), respectively, of the samples are expected positive based on the regression results

Focusing on the individual Omicron sublineages from 2022 and 2023, the LoD analyses revealed that CLEIA was up to sevenfold (LoD50) and up to twofold (LoD95) inferior regarding the detection of BQ.1-positive samples compared to the other subvariants. In contrast, ELISA was up to sixfold (LoD50) and ECLIA up to 36-fold (LoD50) superior in scoring XBB.1.5-positive samples positive, respectively (Fig. 5; Table 5). However, it must be stated that for these lineage subgroups the sample numbers were very small limiting the conclusions to be drawn.

**Table 5:** Summary of non-synonymous amino acid substitutions with a prevalence of > 75% in the nucleocapsid protein of the SARS-CoV-2 VoCs examined in this study in publicly available VoC sequences compared with the original Wuhan-hu-1 sequence. Source: https://outbreak.info/situation-reports

Amino acid position	P13	DEL30/32	DEL31/33	S33	D63	E136	R203	G204	G215	D377	S413
Delta (B.1.617.2)					G		M		C	Y	
Omicron-BA.1.1	L		del				K	R			
Omicron-BA.2	L		del				K	R			R
Omicron-BN.1	L		del				K	R			R
Omicron-XBB.1	L		del				K	R			R
Omicron-BA.5	L		del				K	R			R
Omicron-BQ.1	L		del			D	K	R			R
Omicron-BF.7	L	del		F			K	R			R

del: deletion

### Reevaluation of the measurement kinetics of the automated antigen tests for PCR-negative samples

It is striking that for the Omicron samples from 2023 the ECLIA achieved a significantly higher sensitivity than in the previous cohorts. We, therefore, compared the measurement behavior of the ECLIA with the ELISA and CLIA, as they showed similar test kinetics (Fig. 2). The CLEIA had an extremely broad dynamic measurement range that continued much further below the cutoff than the CLIA, ELISA, and ECLIA. Therefore, CLEIA was excluded from the following analysis. To obtain further information on characteristics of ECLIA, the measurement results of the 303 PCR-negative samples from the non-Omicron cohort were re-analyzed [32]. Using the manufacturer's recommended cutoffs, the mean relative deviation from the cutoff for the ECLIA measured values was -22.59%, which is only 1.91 times the standard deviation. For CLIA and ELISA, the mean deviations from the cutoff were significantly higher at -53.98% (8.25-fold standard deviation) and −56.88% (4.28-fold standard deviation), respectively.

### Comparable detection of tissue culture-expanded VoCs by quantitative, automated SARS-CoV-2 antigen tests

**Fig. 6:**
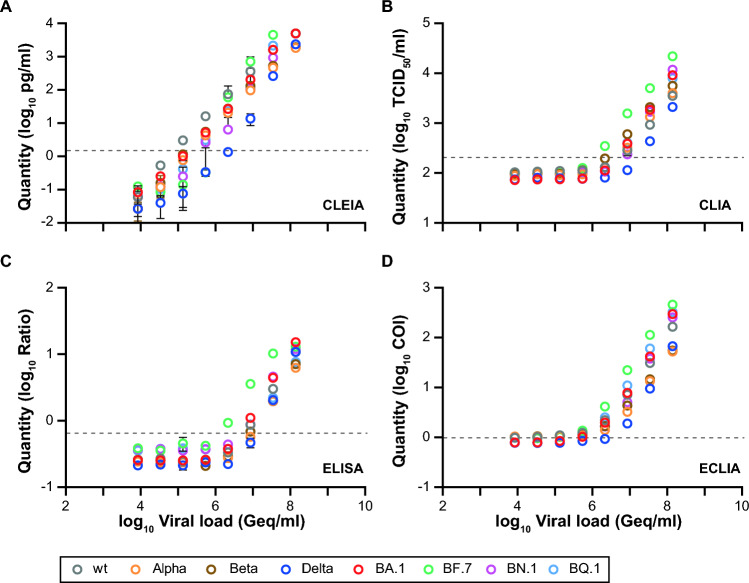
Evaluation of VoCs isolated in cell culture experiments using the (semi-)quantitative SARS-CoV-2 Ag tests: **A** CLEIA, **B** CLIA, **C** ELISA and **D** ECLIA. The measured nucleocapsid protein results were plotted against the calculated SARS-CoV-2 RNA loads**.** The grey dashed line indicates the cutoffs recommended by the manufacturers

In our final approach to characterize the performance of the four automated Ag tests, we extended our analyses to tissue culture-expanded Delta, Omicron-BA.1, -BF.7, -BN.1, and -BQ.1 clinical isolates (Fig. 6A-D). While all of these expanded VoCs could be detected by these four tests, their performance differed widely: for CLEIA, the investigated VoCs scored positive with concentrations ranging between 135,000 Geq/ml (wt and BN.1) to 2,170,000 Geq/ml (Delta and BF.7). Both for CLIA and ELISA, higher concentrations between 2,170,000 Geq/ml (BF.7) and 34,645,828 Geq/ml (Delta) were necessary, similar to observations made for patient swab specimens. When analyzing the ECLIA, we noticed that the values at low concentrations fluctuated around the manufacturer’s cutoff, especially true for wt, Alpha, Beta and BN.1. Of note, in diluted and inactivated mock cell culture samples, positive signals around the cutoff were also measured, similar to the measurements of the VoCs (data not shown). This indicates that the ECLIA records non-specific, low positive signals with this type of culture medium. For the ECLIA, samples were scored positive with concentrations ranging from 135,000 Geq/ml (BQ.1, BN1, BF.7) to 8,660,000 Geq/ml (Delta).

## Discussion

This study greatly expands our previous investigation [32] on the evaluation of four automated quantitative SARS-CoV-2 Ag tests, which detect the nucleocapsid protein and are used in the European market for the quantitative detection of SARS-CoV-2 variants in a diagnostic laboratory setting. Our main observation is a pronounced heterogeneity of these test systems examined for the detection of SARS-CoV-2 VoCs.

The Omicron-positive specimens tested in the current study differed from the historical “non-Omicron” specimens [32] by having significantly higher viral loads on average (Fig. 1C). This may either be due to facilities and test centers, from which the samples were obtained (submission to Max von Pettenkofer Institute exclusively from either clinical units of hospitals (first study) [32] or submissions to Labor Becker MVZ GbR from multiple sources, current study, see also Methods) or to generally higher viral loads in Omicron-infected individuals, as reported by others [60–62]. Consequently, we compared the three cohorts considering this altered distribution of viral loads by Ct/Cp-based stratification of results or by logistic regression modeling as well as calculation of diagnostic sensitivities.

The heterogeneity of the diagnostic sensitivities of the four automated Ag tests for Omicron-positive samples in this study was in the same order of performance as for the previously reported “non-Omicron” samples [32], although the true-positive rates were lower for the Omicron samples. An exception are the measurements of the Omicron samples from 2023 using ECLIA, which will be discussed below. In addition, the differences in sensitivity between the assays changed when a cutoff was used that set specificity at 97%, the minimum criteria recommended by the WHO for RATs (Tables 3, 4). Comparison of these analyses confirmed the recommendation of many manufacturers who encourage performing laboratories to evaluate the most appropriate cutoffs. The ROC analyses presented for Omicron-positive specimen were based on specificity data obtained in previous measurements [32]. Due to the underrepresentation of low viral load samples, the curves for the Omicron-containing specimen shifted upward. The effect was even more pronounced in the Omicron cohort from 2023 than in the one from 2022. This resulted in a very high AUC of 0.993 for ELISA for Omicron samples from 2023 (Fig. 3). However, it was not equally pronounced for all automated Ag assays, and the overall shape of the ROC curve for CLIA, for example, was still parallel to the diagram's diagonal. The comparison of the regression analyses and the examination of the LoD values in both specimen groups showed differences between the Omicron-containing samples from 2022 and 2023, compared to “non-Omicron”-containing samples that did, however, not reach statistical significance. This result appears to be independent of the mean viral load of the two cohorts, as the detection limit of the nucleocapsid-based assays was significantly higher than that of the screening PCR used for sample identification. For Omicron-positive samples, the calculated LoD50 values of the four automated Ag tests ranged from 44,444 to 1,866,900 Geq/ml for Omicron (2022) and from 23,019 and 1,152,048 Geq/ml for Omicron (2023), while the values for “non-Omicron” samples were lower, ranging from 6181 to 749,792 Geq/ml. Due to the high confidence intervals, these differences were not significant. We conclude that the analytical sensitivity of automated Ag test underlies a considerable test system-dependent variability and a trend for a reduced ability of these assays to detect Omicron-containing respiratory samples is observed in regression analyses. Although the analyzed subgroups of different Omicron sublineages from 2022 and 2023 were small, and therefore, statistical evaluations were of limited value, no trend of reduced sensitivity in automated Ag testing can be identified. Moreover, we confirm [32] that the analytical sensitivity of these quantitative automated Ag test is not generally superior to RATs used in a POC or at home setting [24, 47, 63]: In a comparative study on VoC detection by nine RATs we demonstrated an impaired recognition of respiratory samples containing Omicron-BA.1 compared to Delta in seven out of nine tests [24] and these differences reached statistical significance for four of these RATs. In another recent study examining five different RATs for their performance in detecting Omicron-BA.1 and BA.2 [47], we also demonstrated that at high viral loads these RATs achieved higher sensitivities for Omicron-BA.1 than for BA.2, although this trend did not reach statistical significance. One reason for the somewhat less pronounced VoC dependence in performance of automated Ag assays might be differences in the extraction or inactivation buffers used: RATs are believed to operate with rather mild detergents and thus virus particles or aggregates contained in the swab specimen may not be completely inactivated or disaggregated [64, 65]. This is because there is a potential danger of harming layperson users outside of a diagnostic laboratory by chemicals contained in the kits. We speculate that the overall accessibility of the nucleocapsid protein present in a clinical specimen during RAT testing could be lower compared to laboratory-based assays. In addition, first clinical studies evaluating VoC-dependent performance of RATs in a POC setting [46] support the hypothesis that not only the amount of nucleocapsid protein present in the swab, but also pre-existing (partial) immunity may have an influence on the sensitivity of Ag testing. The exact composition of extraction buffers is typically not disclosed by the manufacturers; however, their handling by trained laboratory personnel with personal protective equipment may allow the use of buffers with stronger denaturing or disaggregating conditions.

A limitation of our study is that despite the prospective study design not all manufacturer's recommendations regarding swab kit/transport media and sample storage conditions could be followed. According to available manufacturer information, two of the automated Ag tests investigated here are not based exclusively on one detecting monoclonal antibody, but on polyclonal anti-nucleocapsid antibodies (CLIA) or a mixture of different monoclonal antibodies against different epitopes of the nucleocapsid protein (CLEIA). Usually, RATs only use a single monoclonal antibody for detection. An advantage in sensitivity of CLEIA and CLIA by this multi-epitope approach was, however, not apparent in our study. Conceptually, these multi-target Ag detection assays may be less prone to underperformance upon emergence of new point mutations in the nucleocapsid protein of SARS-CoV-2. In general, newly emerging VoCs should be continuously evaluated to determine whether they have an impact on the sensitivity of RATs and automated Ag tests. Ideally, a transfer of information from manufacturers to diagnostic laboratories and regulatory health authorities is desirable when antibodies used in test devices may bind to regions in the nucleocapsid protein of SARS-CoV-2 expected to impact the performance of Ag tests for detecting new and future VoCs.

During the second study phase 2023, the sensitivity of ECLIA was higher at low viral loads compared to Omicron samples from 2022 and “non-Omicron” samples. On closer inspection of the measurement kinetics, the diagram of Fig. 2D showed a trend of the respective readings towards an asymptote close above the cutoff of 1.0. We can only speculate about the reason for this discrepancy, but tend to view the measurements collected for the ECLIA in 2023 with caution and not overestimating them for the following reason: Our analysis of test kinetics for PCR-negative samples below the cutoff demonstrated distinct differences between the four tests investigated. The CLEIA stands out from the other tests with only about half of the 303 PCR-negative samples lying on the lower asymptote/lower limit of the measurement range (0.01 mg/ml; data not shown). The other three tests, including the ECLIA, did not show such a dynamic in this measurement range. Analysis of the mean deviation of the measured values of the PCR-negative specimens from the cutoff clearly demonstrated that the lower asymptote of the measuring range for ECLIA was only slightly below the cutoff with less than two standard deviations. For CLIA and ELISA, at least four standard deviations of the measured values of PCR-negative samples from the cutoff have been established by the manufacturers for unambiguous differentiation. The small difference in the measured values of low-positive specimens, which lie at the lower asymptote of the ECLIA, from the cutoff is so small that we cannot exclude the possibility of shifts in the results of low-positive specimens above the cutoff in different series of measurements. According to the manufacturer, no changes were made to the commercial assay between 2022 and 2023. A further confounding factor could be batch-dependent fluctuations. Due to the limited amount of material, it was unfortunately not possible to repeat measurements to explore this. When using cell culture media-derived VoC samples in this study, the ECLIA was susceptible to interference from the medium used resulting in low false-positive measurements. However, since the VTM used for the 2023 Omicron samples was not changed according to the supplying laboratory, in contrast to the 2022 samples, it is unlikely that an interfering transport medium is the cause of the observed differences in the evaluation of the patient samples.

In the current phase of the pandemic, different Omicron sublineages or recombinants co-evolve regionally. Originating from previously dominating variants, subvariants derived from Omicron-BA.2 (BN.1, BA.2.75), BA.2 recombinants (XBB) or BA.5 subvariants (BQ.1, BF.7) have emerged. Besides additional adaptions in the spike protein, some of these have also acquired mutations in the nucleocapsid protein (e.g., BF.7: Del30/32, S33F, or BQ.1: E136Q [66]) (Table 5). A broad understanding of the complex interplay between molecular changes caused by mutations, interference with the host immune system and the impact of the latter on disease progression, virus production and release onto the respiratory mucosa is essential for the continued use of Ag-based diagnostic methods with appropriate reliability. We strongly believe that both RATs and automated Ag tests should be carefully re-evaluated by independent laboratories when new VoCs emerge or immunological and clinical parameters in the population change.

## Data Availability

The datasets generated and analyzed during the current study are available upon request.
